# Optimized Super-Wideband MIMO Antenna with High Isolation for IoT Applications

**DOI:** 10.3390/mi13040514

**Published:** 2022-03-25

**Authors:** Adnan Khurshid, Jian Dong, Mir Swad Ahmad, Ronghua Shi

**Affiliations:** School of Computer Science and Engineering, Central South University, Changsha 410083, China; adnan.khurshid1987@gmail.com (A.K.); mirswadahmad@gmail.com (M.S.A.); shirh@csu.edu.cn (R.S.)

**Keywords:** MIMO (multiple-input–multiple-output), wireless communication, SWB (super-wide-band), envelope correlation coefficient, diversity, isolation

## Abstract

A compact, low profile, multiple-input–multiple-output (MIMO) diversity antenna with super-wideband (SWB) characteristics has been proposed. The proposed antenna comprises four symmetric monopole-radiating elements printed on low-cost FR4 substrate with the slotted ground plane. The single antenna of a monopole structure and a quad-port MIMO antenna, with the dimensions of 30 × 20 mm^2^ and 60 × 55 mm^2^, respectively, are ideal for IoT and high-speed data applications. The proposed MIMO antenna has a high diversity gain and low envelope correlation coefficient (ECC) within the frequency range. Simulated results demonstrate the performance of the MIMO-SWB antenna, which operates from 2.3 to 23 GHz, with a high isolation level over 20 dB in the achieved frequency band. Moreover, the proposed MIMO antenna has been investigated with mirror fashion and orthogonal structure. Both structures provide similar results except for mutual coupling performance. The orthogonal adjustment for high isolation achieves better results with the proposed model. Further, the prototype of the proposed antenna is fabricated and measured effectively. Simulated and measured results show good agreement for super-wideband applications.

## 1. Introduction

To achieve a higher data rate and reliable services, super wideband is the most preferred technology for today’s wireless communication. It has promising applications in short-range transmission, indoor, and medical applications. By expanding bandwidth, we can leverage marge wireless technologies in one device for more better product [1]. Ultra-wideband (UWB) operating band is from 3.1 to 10.6 GHz for wireless applications [2]. For SWB, there is no predefined frequency spectrum, such as UWB. Comparison in terms of the wideband antenna is to perform in terms of bandwidth ratio, which should be at least 10:1. MIMO was introduced to improve multiplexing gain and diversity to improve link quality [3]. To control multipath fading, we need to place multiple antenna elements in a limited area, which causes distraction because of mutual coupling [4]. To control mutual coupling, many techniques were explored in the literature. So far, many designs and methods have been introduced by researchers for expanding bandwidth and enhancement of MIMO technology for system stability. However, the problem of super wideband with high isolation in the complete spectrum within a compact structure is still serious.

SWB-MIMO antenna performance depends on the design structure. MIMO antenna has many design challenges to achieve wide bandwidth, high isolation, radiation pattern, stable gain, and size reduction. To overcome these challenges, MIMO antenna is examined by many researchers, and a variety of techniques are adopted, such as defected ground structure, metamaterial, feeding structure, neutralization line, isolation based on pin diode, and parasitic element approach. Defected ground disturbs the surface current, as a result, reduces mutual coupling among antenna elements [5]. The neutralization line approach is used to solve the problem of antenna matching and improves the isolation between antenna elements [6,7]. The etching Slot technique controls the current on-ground plane by suppressing the coupling between the closely spaced antennas and acts similar to a band-stop filter [8]. Similarly, for negative permittivity/permeability effects, metamaterial structures can be placed in radiation patch, ground, or between the antenna elements [9].

The concept of MIMO technology combined with SWB is an exciting research topic for enhancing today’s wireless communication system [10,11,12]. For example, 5G super-high bit rate transmission supports simultaneous operation of low-power sensors and high-definition video streaming. In [13,14,15,16], simple SWB antennas in the planar and coplanar domains were presented from the literature. In [17], the author presented a SWB-MIMO antenna with size reduction technique by an appropriate alteration in the feedline using a multistage numerical optimization method. Furthermore, in [18,19,20], all authors proposed MIMO antennas with good mutual coupling and suitable diversity gain. However, some of these antennas are miniaturized in size but compromised on expanding bandwidth from UWB range or minimum antenna isolation and correlation coefficient in the whole frequency band. In [21,22,23,24,25,26,27], the SWB-MIMO technique was implemented in the planar and coplanar domain. The parasitic structure was used to reduce mutual coupling in the monopole planar structures [28,29]. Although these MIMO antennas achieved super wideband and high isolation, they are still large for high data-rate transmission applications. Despite these designs, we still need a low-profile, compact SWB-MIMO antenna for high-data-rate applications. In IoT applications, advance wireless technologies are merging to introduce new features in one device [30,31].

Hence, the research intends to design low-profile planar SWB-MIMO antennas with good performance of isolation, gain, ECC, and efficiency. The proposed structure can perform as single, two-port, and four-port antenna on the same operating frequency range from 2.3 to 23 GHz. Furthermore, the orthogonal adjustment is used to improve isolation. The proposed antenna model with its performance and characteristics is discussed in the following section.

## 2. Antenna Design and Methodology

### 2.1. Antenna Geometry

**Figure 1:**
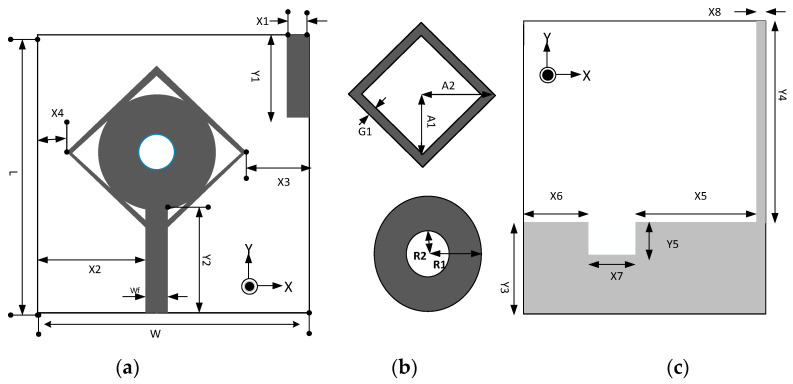
Geometry of the proposed single-element antenna. (**a**) Front view, (**b**) radiation patch elements, and (**c**) back view.

**Figure 2:**
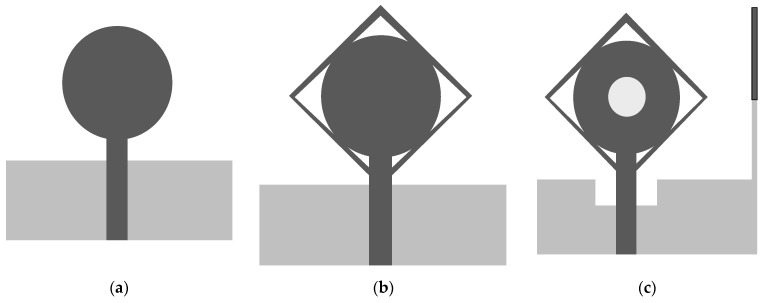
Antenna investigation analysis (**a**) Type I, (**b**) Type II, and (**c**) Type III.

**Figure 3:**
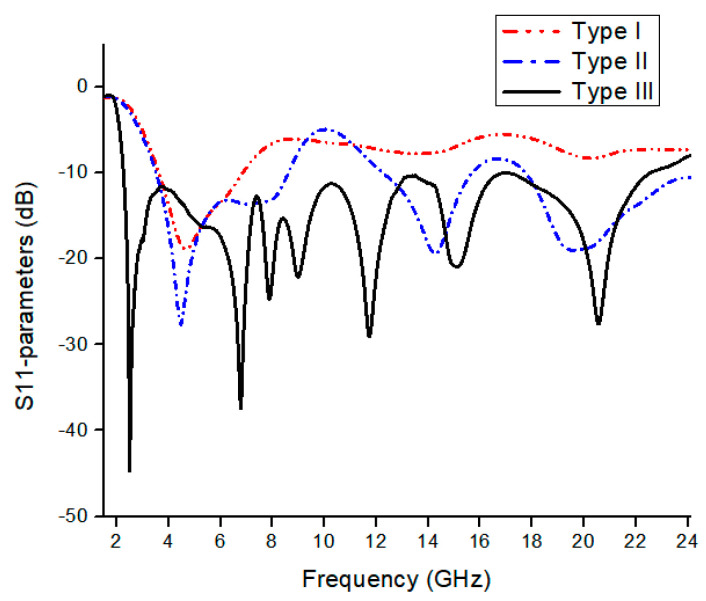
Reflection coefficients of the design phases.

A single element antenna with SWB is shown in Figure 1. Antenna fabrication is performed on the FR4 substrate (*ε_r_* = 4.3, tan δ = 0.02) with a thickness of 1.2 mm. Monopole structure introduced for SWB, feedline width W_f_ is 1.5 mm to achieve 50 Ω characteristic with the dimension of 30 × 20 mm^2^. The dimensional details of the antenna resonating element are presented in Table 1. The development and optimization of the antenna was performed by using the CST MWS. The SWB antenna was investigated on three different design stages: Type I, Type II, and Type III, which can be observed from Figure 2. Type I is derived from a conventional circular monopole antenna. The circular shape was used in many UWB antennas to find wideband results. A circular radiation patch integrated with a square ring forms type II to achieve wide bandwidth. By etching in the radiation patch and ground plane with the additional stub, we achieved type III. Three stage design structures are shown in Figure 2. The initial antenna performed poorly; some significant contributions have been made in Type I to meet desired results. Type II structure gives bandwidth on the higher frequencies, which helps to expend the bandwidth. For better results, etching in the ground plane significantly improves performance. The reflection coefficient of the three stage designs is shown in Figure 3. The proposed antenna is optimized through simulation, exhibiting a bandwidth of 2.3 to 23 GHz.

**Figure 4:**
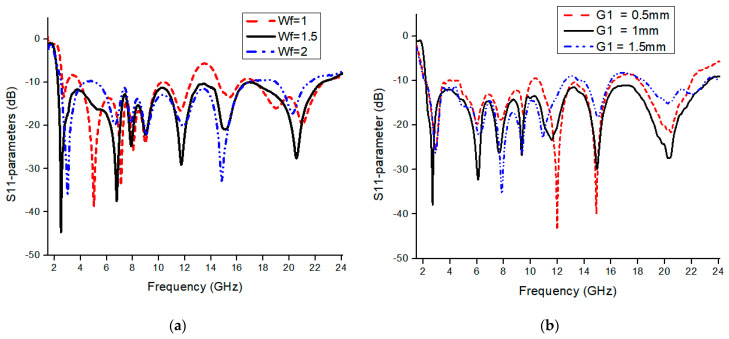
Simulation response of (**a**) feedline and (**b**) square ring.

**Figure 5:**
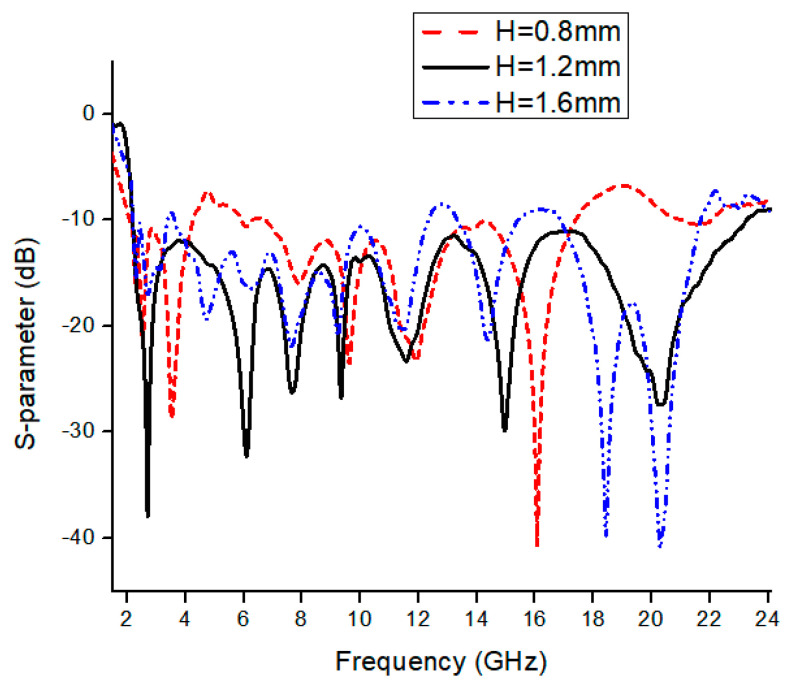
Simulation response of substrate height.

**Figure 6:**
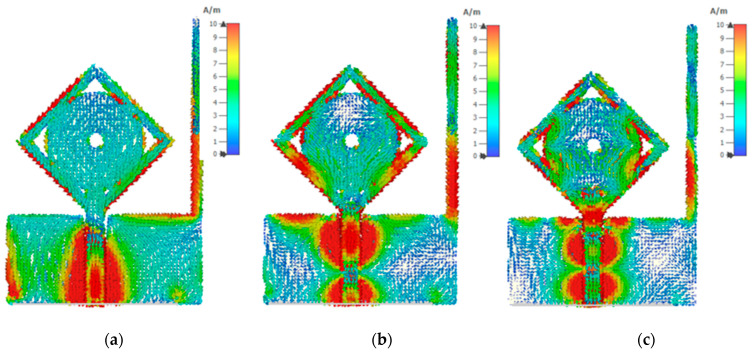
Surface current distribution of the antenna at (**a**) 5 GHz, (**b**) 12 GHz, and (**c**) 20 GHz.

Design optimization is started by the investigation of a single monopole antenna. The single antenna element was optimized to achieve the same results for MIMO systems (two-port and four-port antenna). While investigating the type I model, the circular radiation patch was introduced with the square ring. The addition of the square ring improves frequency bandwidth. Then, subtracting the center portion of the circular radiation patch current distribution was disturbed, which increased the effective inductance and capacitance for minimizing the lowest operating frequency from 3.6 to 2.3 GHz. To find the effective area of the radiation patch, the empirical formula for the lower cut-off frequency is used for a planar monopole antenna [32,33].

(1) $$A=\pi *r^{2}\;\;,\;\;A=a^{2}$$

(2) $$C=2*\frac{A}{r}\;\;,\;\;\varepsilon_{eff}\approx \frac{\varepsilon_{r}+1}{2}$$

(3) $$f_{L}=\frac{c}{\lambda }\;\Rightarrow \;\frac{c}{2*C\sqrt{\varepsilon_{eff}}}\mathrm{GHz}$$

where *r* is the radius and *C* is the circumference of the circle, *ε_eff_* is the effective dielectric of the patch. We can drive the lower cut-off frequency from the above formulas, as we observe from simulation that proposed antenna *f_L_* is 2.3 GHz, which is also approximately equal through the above equation. To analyze the antenna performance, parametric analysis is performed to optimize the dimensions of different parameters. To calculate the dimension of the feedline, we analyze it through a simulation process. We find the best results on 1.5 mm feedline width as described in Figure 4a that the −10 dB bandwidth is achieved in an entire frequency band. For SWB, the square ring performed a vital role in expanding the antenna’s bandwidth. Figure 4b shows the impact of the square ring width on the reflection coefficient. If we increase the width of the square ring, the bandwidth range compromises on higher frequencies, and by decreasing the width of the square ring, it bring off 10 dB from 8 GHz to 11 GHz considering all other parameters fixed. Figure 5 demonstrates the substrate height optimization. It was observed from the simulation that substrate height has a significant effect on the results of the high frequencies. Frequency bandwidth was compromised from 12 GHz to 18 GHz to achieve 10 dB reflection coefficient at the entire frequency band but achieved better impedance matching results from 18 to 21 GHz by increasing the substrate height. By decreasing the substrate height, frequency bandwidth is compromised to achieve 10 dB at low frequencies. It is also known that the bandwidth is generally proportional to substrate thickness and inversely proportional to substrate permittivity. Figure 6 shows the current distribution effect at three different frequencies. Current density is maximum on edges of the radiation patch, feedline, and center of the ground plan. The current concentration is mainly on the bottom of the feedline, radiation patch edges, and ground plane at low frequencies. By moving at high frequencies, the current distribution is mainly on the edges of the radiation patch, varying slightly upwards on feedline and center of the ground plan. At higher frequencies, ground plane stub only shows current on internal edges, it helps in MIMO structure to improve isolation between the radiating elements.

### 2.2. SWB Antenna with Four Ports

**Figure 7:**
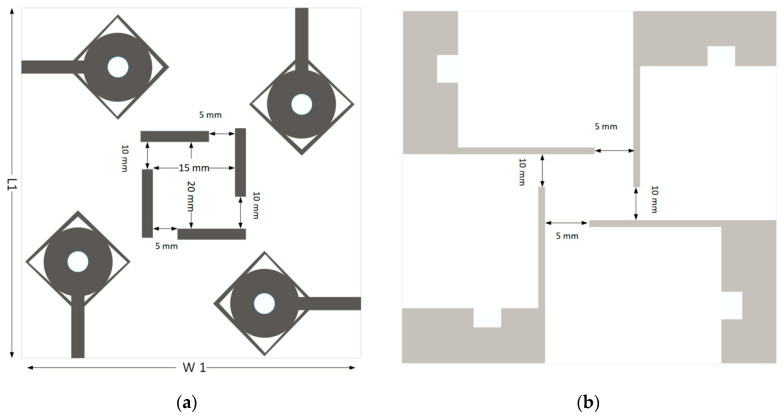
MIMO SWB antenna. (**a**) Radiation patch and (**b**) ground plane.

**Figure 8:**
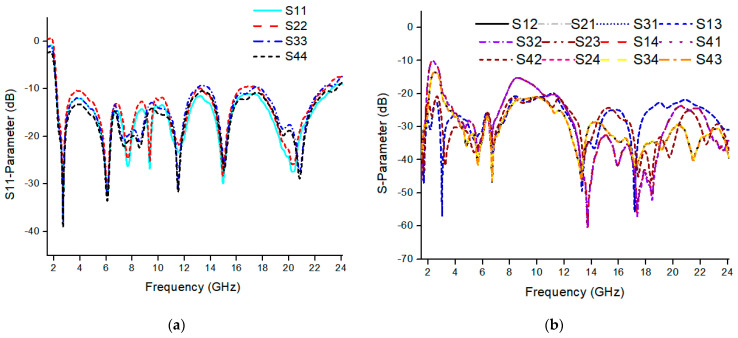
Simulation response of the orthogonal MIMO antenna. (**a**) S-parameters and (**b**) mutual coupling.

The geometry of the proposed SWB-MIMO antenna system is shown in Figure 7, and antenna elements are arranged orthogonally. MIMO radiation element and ground plane are the same as defined in the single antenna. The size of the proposed SWB-MIMO antenna is 60 × 55 × 1.2 mm^3^, where L1 is 60 mm and W1 is 55 mm. Compared with single and two-port MIMO antennas, a four-port MIMO antenna design is more critical due to the mutual coupling of antenna elements. The basic antenna model was designed in such a way that it controlled mutual coupling between antenna elements with the ground stub, and for high isolation, antenna elements are arranged orthogonally in adjustment. The proposed MIMO antenna is first analyzed with a mirror fashion structure. The width of the MIMO antenna was reduced by 15 mm (i.e., 60 × 40 × 1.2 mm^3^) but the isolation level was lower than the orthogonal structure. The simulated scattering parameters of the SWB-MIMO antenna are shown in Figure 8a. Furthermore, it shows that an isolation level higher than 20 dB is achieved over 90% of the entire frequency range, as shown in Figure 8b. The MIMO antenna shows good agreement in reflection coefficient with the single antenna model.

#### Diversity Analysis

**Figure 9:**
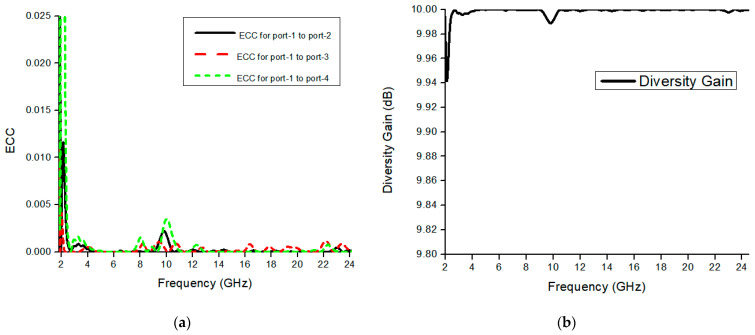
Simulation response of the orthogonal MIMO antenna. (**a**) ECC and (**b**) diversity gain.

The envelope correlation coefficient is used to check the second port’s effect on the first port for diversity analysis. The acceptable limit for the ECC is ECC < 0.5, obtained ECC of the three-element MIMO antenna shown in Figure 9a, which is less than 0.002 throughout the band. The correlation between MIMO elements is small, which illustrates the SWB-MIMO antenna has very good diversity performance. ECC calculates the diversity gain according to the formula below [24].

(4) $$\mathrm{Gian}=10*\sqrt{1-\left|\mathrm{ECC}\right|}$$

The diversity performance of the proposed SWB-MIMO antenna is illustrated in Figure 9b. The diversity gain of the MIMO antenna is almost nearly 10 dB within the frequency range knowing that it starts from 9.94 dB at 2 GHz, as shown in the figure.

## 3. Results and Discussion

**Figure 10:**
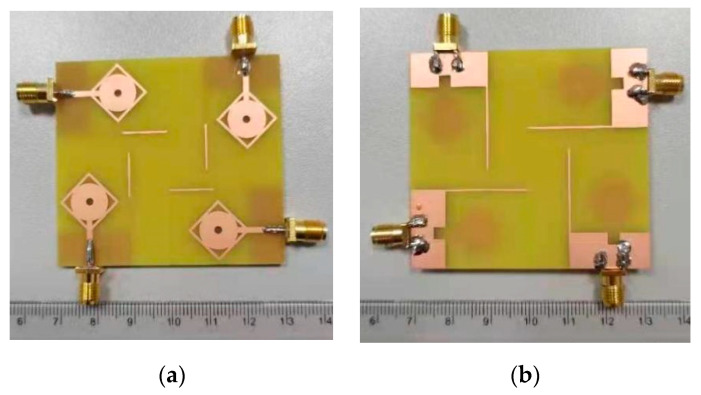
Fabricated prototype of MIMO antenna. (**a**) View from the top and (**b**) view from the bottom.

**Figure 11:**
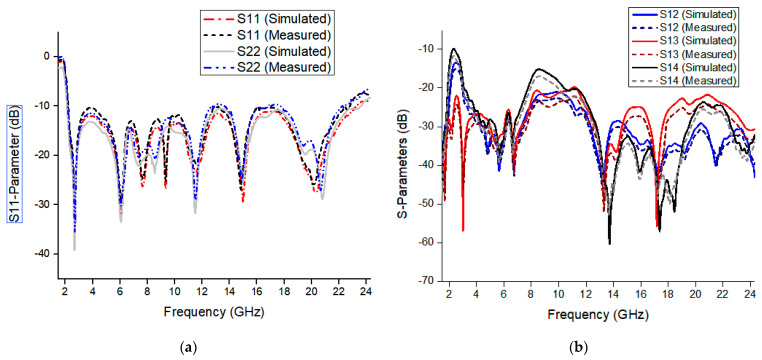
Measured and simulated S-parameters of the proposed MIMO antenna with respect to port1. (**a**) S-parameters and (**b**) isolation.

**Figure 12:**
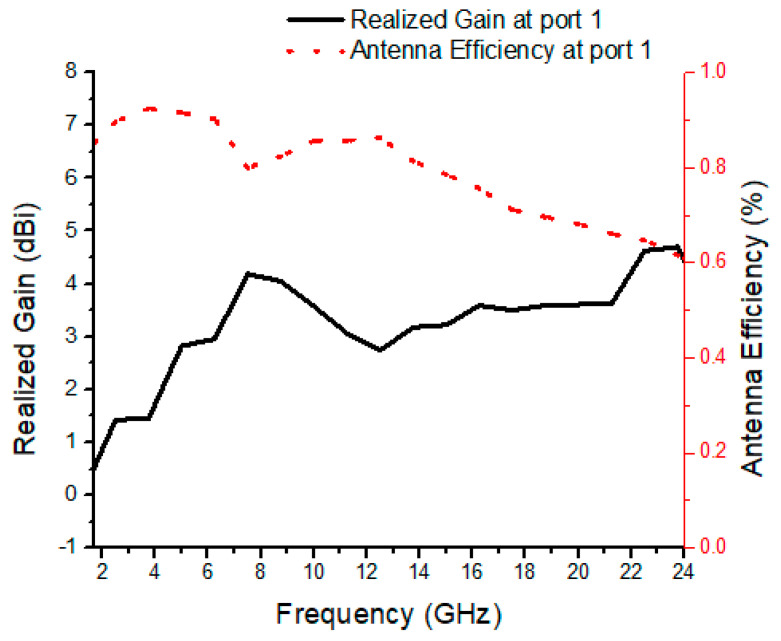
Measured realized gain and efficiency of the MIMO antenna port 1.

The fabricated prototype of the proposed antenna is shown in Figure 10. Agilent E8364B PNA analyzer is used to measure its performance. The antenna contains four monopoles in the orthogonal arrangement. The radiation patch of the antenna is connected with a 50 Ω feedline, which is in turn connected to match the 50 Ω SMA connector for antenna characterization for both fields. There are strong correlations between the measurement and simulation results of the prototype. Figure 11 shows the measured and simulated results of the reflection coefficient and mutual coupling of the proposed antenna. The frequency bandwidth of less than −10 dB is 2.3 to 23 GHz, which is appropriate for the requirement of reflection coefficient for indoor application, vehicle sensor, and high data-rate transmission. The isolation level of the proposed antenna is over 20 dB. Figure 12 shows the measured realized gain and antenna efficiency. The maximum antenna gain is 4.5 dBi and antenna efficiency ranges from 60% to 90% within the whole band.

**Figure 13:**
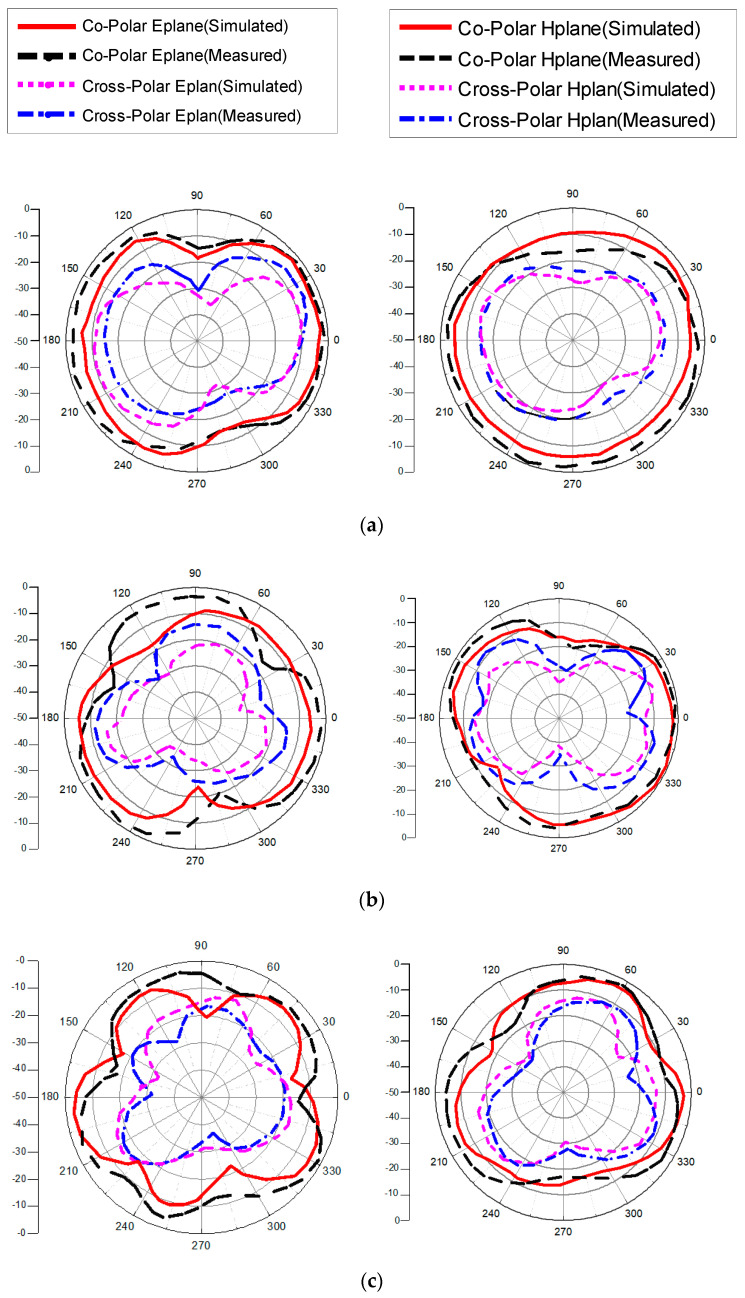

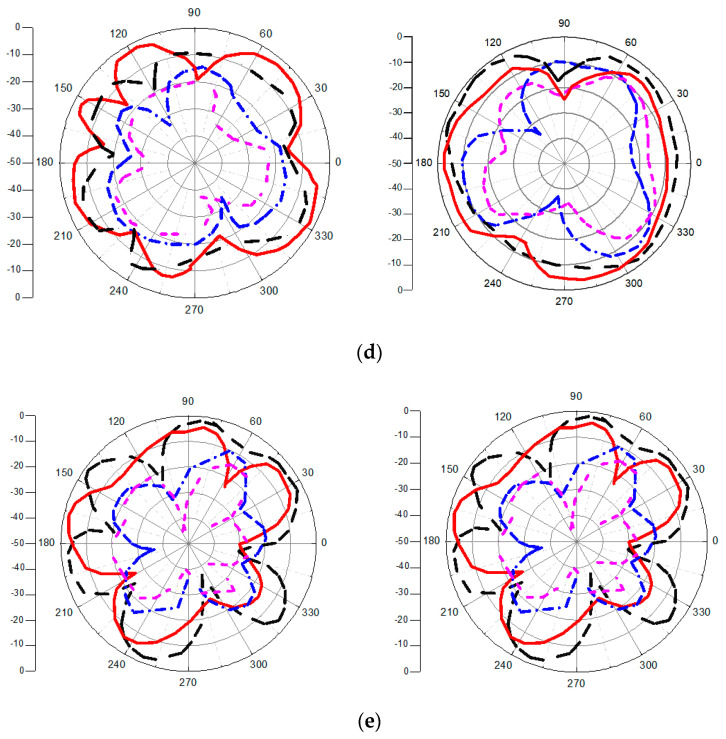
Simulated and measured response of co- and cross-polar radiation of the MIMO antenna on port 1 at the frequencies (**a**) 3.5 GHz, (**b**) 8 GHz, (**c**) 12 GHz, (**d**) 16 GHz, and (**e**) 21 GHz.

The radiation properties of the proposed antenna are illustrated in Figure 13. The simulated and measured radiation characteristics for the E-plane (yz-plane) and the H-plane (xz-plane) are analyzed at different frequencies on port 1. The proposed MIMO antenna provides quasi-omnidirectional characteristics on co-polarized fields in E-plane and cross-polarization fields in H-plane because current distribution is uniform between ground plan and radiation patch at all frequencies. From Table 2, the comparison shows the proposed antenna is a suitable candidate with achieved characteristics. From the literature, it is observed by expending bandwidth other parameters are compromised in MIMO system. The comparison shows high isolation in [17], but its bandwidth is less than the proposed antenna. Although it has a compact structure, it only has two ports. In [22], SWB with high isolation is presented, but the proposed antenna has much better ECC than the design in [22]. The antennas in [25,26] have larger bandwidths than the proposed antenna, but the isolation and size is better in the proposed antenna.

## 4. Conclusions

This paper presents a novel SWB-MIMO antenna with high isolation, wide frequency bandwidth, and low ECC. To achieve the SWB, a square ring is placed within the radiation patch, which improves the impedance bandwidth. An orthogonal structure for a four-element SWB-MIMO antenna system is ideal for a high data-rate transmission. The operational frequency ranges from 2.3 to 23 GHz for single and multi-elements. A decoupling structure in the ground plane is introduced with an orthogonal adjustment to improve port isolation as high as over 20 dB. The simulated and measured results demonstrate quasi-omnidirectional radiation patterns, strong isolation, and minimum ECC. The proposed SWB-MIMO antenna system can potentially be used in high-speed data communications, IoT, and short-range communication systems.

## Figures and Tables

**Table 1:** Proposed antenna dimension.

Parameter	Dimension (mm)	Parameter	Dimension (mm)
L	30	Y5	3
W	20	R1	5
X1	0.5	R2	1
X2	8.25	G1	1
X3	3	A1	7
X4	1	A2	8
Y1	12	X5	9
Y2	12.1	X6	7.5
Y3	9	X7	3
Y4	21	X8	0.5

**Table 2:** Comparison of the proposed MIMO antenna with other reported antennas.

Ref.	Bandwidth		Dimensions		Isolation (dB)	ECC
	GHz	Ratio	mm^2^	*λ*_L_ × *λ*_L_ (Lower Frequency)		
[2]	3–18	6:1	40 × 40	0.30*λ* × 0.30*λ*	>20	<0.03
[17]	2.8–20	7.1:1	17.7 × 30.7	0.13*λ* × 0. 23*λ*	>22	<0.005
[18]	3.2–11	3.4:1	36 × 36	0.27*λ* × 0. 27*λ*	>15	<0.005
[19]	2–10.6	5.3:1	45 × 45	0.34*λ* × 0. 34*λ*	>17	<0.005
[20]	3–15	5:1	38 × 38	0.29*λ* × 0. 29*λ*	>20	<0.005
[21]	2.9–40	13.7:1	58 × 58	0.44*λ* × 0. 44*λ*	>17	<0.04
[22]	1.3–40	30.7:1	56 × 56	0.43*λ* × 0. 43*λ*	>22	<0.03
[23]	2.24–30	13.3:1	45 × 90	0.34*λ* × 0. 69*λ*	>10	N-A
[24]	3.5–4.4, 6–20	4.5:1	67 × 67	0.51*λ* × 0.51*λ*	>20	<0.05
[25]	0.96–35	36:1	63 × 63	0.48*λ* × 0.48*λ*	>17	<0.01
[26]	1.3–40	31:1	63 × 63	0.48*λ* × 0.48*λ*	>16	<0.05
[27]	3.1–20.3	6.4:1	50 × 50	0.38*λ* × 0.38*λ*	>20	<0.05
[28]	3–40	13.3:1	18 × 36	0.13*λ* × 0.27*λ*	>18	<0.01
[29]	3.8–51	13.3:5	31 × 31	0.23*λ* × 0.23*λ*	>15	<0.075
Proposed	2.3–23	10:1	60 × 55	0.46*λ* × 0.42*λ*	>20	<0.002
